# Self-administered active versus sham acupressure for diarrhea predominant irritable bowel syndrome: a nurse-led randomized clinical trial

**DOI:** 10.1186/s12912-024-02594-5

**Published:** 2025-01-28

**Authors:** Maha Gamal Ramadan Asal, Ahmed Abdelwahab Ibrahim El-Sayed, Samira Ahmed Alsenany, Zahraa Hassan Ramzy, Rasha Fathy Ahmed Dawood

**Affiliations:** 1 Medical Surgical Nursing Department, Faculty of Nursing, Alexandria University, Alexandria, Egypt; 2 Nursing Administration Department, Faculty of Nursing, Alexandria University, Alexandria, Egypt; 3 Public Health Department, Faculty of Nursing, King Abdulaziz University, Jeddah, Saudi Arabia; 4 Medical Surgical Nursing Department, Faculty of Nursing, Alexandria University, Alexandria, Egypt; 5 Medical Surgical Nursing Department, Faculty of Nursing, Alexandria University, Alexandria, Egypt

**Keywords:** Active acupressure, Sham acupressure, Irritable bowel syndrome, Diarrhea, Randomized Controlled Trial, Nurse led Trial, Nursing Care.

## Abstract

**Background:**

Diarrhea-predominant irritable bowel syndrome (IBS-D) significantly impacts patients’ quality of life, with existing treatments offering limited relief. Self-administered acupressure presents a potential non-invasive, cost-effective treatment option that could alleviate symptoms and enhance health outcomes in these patients.

**Aim:**

This randomized controlled trial aimed to evaluate the effect of active acupressure compared to sham acupressure on primary and secondary outcomes among IBS-D patients.

**Method:**

The study included 63 patients with IBS-D, recruited from Alexandria Main University Hospital, Egypt. Participants were randomized into either an active acupressure group or a sham acupressure group. Both groups underwent two days of training, followed by four weeks of intervention. The active group applied pressure to specific therapeutic acupoints, while the sham group used non-therapeutic points. Outcomes were assessed at baseline, week 2, and week 4.

**Results:**

The active acupressure group showed a significant reduction in symptom severity, improved stool consistency, and frequency, and greater adequate symptom relief by week 4 compared to the sham group. Psychological outcomes, including anxiety and depression, also improved significantly in the active group. Additionally, the active group reported reduced use of rescue medications.

**Conclusion:**

Active acupressure is an effective nursing intervention for alleviating symptoms of IBS-D, particularly when applied consistently over time. It improves both physical and psychological outcomes, offering a valuable non-pharmacological treatment option.

**Implications:**

Nurses can integrate self-administered acupressure into IBS-D care plans, teaching patients this technique to manage symptoms independently, thus enhancing their quality of life (QOL) and reducing reliance on conventional medications. This intervention aligns with holistic nursing care and offers a cost-effective, patient-friendly solution for managing IBS-D.

**Trial registration:**

This study was prospectively registered as a randomized controlled trial in https://clinicaltrials.gov/ Registration Date: January 7, 2023, Registration Number: NCT05702255.

## Introduction

Irritable bowel syndrome (IBS) is a common functional gastrointestinal disorder (FGID) that is defined by recurrent abdominal pain that is related to bowel movements or changes in the frequency and/or form axis illnesses. Globally, IBS is one of the leading reasons for primary care consultations, with prevalence rates ranging between 10 and 15% in the general population [1]. In the U.S.A, IBS accounts for 12% of annual primary care visits, while in the United Kingdom., it leads to 2.4 million general practitioner visits yearly, highlighting its significant impact on healthcare and the need for early diagnosis and management [2].

The diagnosis of IBS is based on clinical symptom assessment and the exclusion of organic disorders [3]. According to the Rome IV diagnostic criteria, IBS is characterized by recurrent abdominal pain associated with at least two of the following symptoms: changes in stool form or frequency. This pain must occur, on average, at least once a week over the past three months, with symptoms persisting for at least six months [4].

IBS is a multifactorial disease with a complex pathogenesis. Several functional alterations (e.g., functional brain alterations, visceral hypersensitivity, alterations in bowel motility and secretory function, and psychiatric illness) and gastrointestinal abnormalities (e.g., immune activation, intestinal microbiota alterations, nerve sensitization, impaired mucosal functions, post-infectious plasticity, altered expression and release of immune and mucosal mediators, and altered gene expression profiles) have been involved in those disorders [5]. Also, environmental factors (e.g., diet, food intolerance, antibiotic use) may have a role. Among psychosocial factors, depression and anxiety are the most frequently seen symptoms. Such factors are involved in the brain-gut axis alteration, leading to the exacerbation of IBS symptoms, the continuation of these symptoms, or abnormal disease behavior [6]. According to the predominant stool type, IBS can be classified into four main subtypes: IBS with constipation, IBS with diarrhea (IBS-D), IBS with mixed bowel habits, and unclassified IBS. Globally, IBS-D accounts for around one-third of all cases with IBS [6]. In the United States, the prevalence of IBS-D is similar, affecting approximately 30% of individuals diagnosed with IBS [2].

IBS places a major burden on healthcare systems; it is associated with increased healthcare costs [2], an abnormal state of life, and work impairment with productivity loss [7, 8]. Also, all domains of the health-related QOL for those patients are greatly impaired [9], which is greatest in those with predominant diarrhea [10]. Intestinal pain in patients with IBS-D is typically accompanied by cramps, mucus in the stool, frequent loose stools, and an urgency that is not relieved by defecation [11]. As a result of these symptoms, those individuals feel ashamed when they have to pass flatus or use the toilets in public or at work. Patients may make maladaptive adjustments, such as activity avoidance, to gain control of various personal and work situations [12].

The therapeutic approaches for these patients should consider the most common symptoms and bowel habits. For IBS-D, conventional therapies include pharmacological treatments such as antibiotics, mixed opioid agonists and antagonists, peripheral opioid agonists, bile acid sequestrant, antagonists of serotonin 5-HT3 receptors, antispasmodics, and antidepressants, while non-pharmacological approaches include lifestyle interventions (e.g., stress reduction, physical activity, and dietary modifications), fecal microbiota transplantation, and microbiota manipulation. In addition, complementary and alternative medicine such as acupuncture, hypnotherapy, relaxation techniques, or herbal remedies are known treatment approaches in the treatment of IBS [13].

Unfortunately, control of symptoms is poor for a considerable number of patients. Thus, it is reasonable that the ineffectiveness or associated side effects of conventional treatment, the poorly recognized pathology, and the psychological elements of IBS have led to the progress of complementary and alternative therapies directed at symptom management [14].

A growing number of patients have recently been receiving complementary and alternative therapies, such as moxibustion, acupuncture, and traditional Chinese herbal remedies [15]. Psychological therapies like cognitive behavioral therapy and meditation are considered safe; however, their specificity of application poses challenges to their use as permanent therapies [13]. These approaches often require individualized treatment plans tailored to specific patient needs, making them less suitable for broad or standardized implementation. Additionally, they rely on access to trained professionals, consistent patient engagement, and adherence over time, which may not be feasible for all patients or healthcare systems. These factors limit their scalability and long-term integration as a primary therapeutic option [16].

Acupuncture and acupressure are therapeutic techniques rooted in traditional Chinese medicine (TCM), both aimed at stimulating specific points on the body, known as acupoints, to balance Qi (vital energy) and blood flow. TCM is an ancient system of healing that operates on the belief that health is maintained through a balanced flow of Qi throughout the body. When the flow of Qi is disrupted, it is believed to lead to illness [17]. According to Chinese medicine theory, illness arises from disharmony in the yin-yang organ system. Therapies like acupuncture and acupressure work to restore balance by stimulating acupoints, which are located along pathways known as meridians. Meridians are thought of as channels through which Qi flows, connecting different parts of the body and ensuring the proper distribution of vital energy [18]. In the case of IBS, the condition is believed to stem from imbalances such as weakness in the PI (spleen) and Wei (stomach), or damage to the Gan (liver), often influenced by emotional stress, diet, and other environmental factors, leading to intestinal dysfunction [15].

While both acupuncture and acupressure target the same acupoints, they differ significantly in their mechanisms and intensity. Acupuncture is an invasive technique that uses fine needles to stimulate deeper tissues, activate central nervous pathways, release neuropeptides, modulate cytokines, and influence the microbiota-gut-brain (MGB) axis. It also induces a localized inflammatory response, promoting blood flow and tissue repair [19]. In contrast, acupressure is a non-invasive technique that applies manual pressure using fingers, hand-held devices, or external tools on the same acupoints. This results in milder stimulation of sensory nerves, improved circulation, endorphin release, and stress reduction. While acupuncture achieves deeper physiological effects, acupressure offers a practical, cost-effective alternative that avoids the limitations of invasiveness and can be self-administered [20].

In many conditions such colitis, gastritis, hypertension, and obesity, acupuncture has been used to alleviate the MGB imbalance [21]. In addition, acupuncture is thought to be a helpful alternative therapy for FGIDs [22]. Manheimer et al. (2012) conducted a meta-analysis revealing that acupuncture showed potential benefits for IBS patients, including improved QOL and symptom control, though the findings were not conclusive due to high risk of bias and methodological limitations in some studies [23].

Nurses play an active role in the care of patients with Irritable IBS by providing essential disease-specific education and alleviating patients’ concerns. They assess the specific symptoms present, determine their severity, and understand their impact on the patient’s life. By being knowledgeable about available treatment options, nurses become crucial information channels and valuable facilitators of a positive practitioner-patient therapeutic relationship. This comprehensive approach helps mitigate the negative effects of IBS and improves overall treatment outcomes for patients [24]. However, the invasive nature, cost, and practitioner-administered nature of acupuncture can be limiting factors in its application. In this context, acupressure emerges as an attractive option. Acupressure can be taught to patients to allow them to perform it by themselves instead of being administered by practitioners [20].

Self-administered acupressure is a cost-effective, non-intensive, and patient-friendly approach [25]. Several studies have demonstrated the effectiveness of self-administered acupressure in the management of several conditions, including insomnia disorder [25], fatigue following traumatic brain injury [26], sleep and anxiety in patients undergoing cardiac surgery [27], chronic low back pain [28], bronchiectasis [29], persistent cancer-related fatigue [30], knee osteoarthritis [31, 32], chronic neck pain [33], allergic rhinitis [34], and occupational cognitive failure in nurses after Covid-19 [35].

Despite advancements in the understanding and treatment of IBS, effective and reliable management options remain limited, especially those that are non-pharmacological. Current research predominantly focuses on dietary changes [36], pharmacotherapy [37], and lifestyle modifications [38], with less emphasis on alternative therapies such as acupressure. Also, available studies do not give emphasis to IBS-D; however, those patients constitute a large percentage of all IBS patients. Moreover, the studies that do explore acupressure often lack rigorous design, making it difficult to draw definitive conclusions about its efficacy. A significant research gap exists in the form of high-quality, randomized controlled trials (RCTs) that can robustly compare the effects of active acupressure (AA) versus a placebo control, such as sham acupressure (SA).

Our RCT addresses this research gap by employing a methodologically sound approach to evaluate the effectiveness of AA versus SA in managing IBS-D. This RCT examined the effect of acupressure on both primary and secondary outcomes of IBS-D. Primary outcomes include symptom severity, adequate relief, stool consistency, and stool frequency. Secondary outcomes include anxiety and depression levels and the use of rescue medications. This study’s significance lies in its potential to provide high-quality evidence that could either validate or refute the therapeutic claims of acupressure for IBS. By using a SA group as a control, the study can isolate the specific effects of the AA intervention, thereby enhancing the reliability of the results. This distinction is crucial for determining whether acupressure can be considered a legitimate complementary therapy for IBS-D.

## Materials and methods

### Aim of the study

This RCT aims to examine the effect of AA versus SA on the primary outcomes (symptom severity, adequate relief, stool consistency, and frequency) and secondary outcomes (anxiety, depression, and use of rescue medications) among IBS-D patients.

### Study hypotheses

#### H1

IBS-D patients receiving AA will report a greater reduction in symptom severity compared to those receiving SA.

#### H2

A higher proportion of IBS-D patients in the AA group will report adequate relief of IBS-D symptoms compared to the SA group.

#### H3

AA will result in more normalized stool consistency and frequency compared to SA among IBS-D patients.

#### H4

IBS-D patients receiving AA will experience a greater reduction in anxiety and depression levels compared to those receiving SA.

#### H5

IBS-D patients in the AA group will have lower use of rescue medications compared to the SA group.

### Design and setting

This is a double-blind (participants, outcome evaluators, and statisticians) sham-controlled RCT that adheres to the Consolidated Standards of Reporting Trials guidelines (CONSORT). The study was conducted at the gastroenterology outpatients’ clinic of Alexandria Main University Hospital, Egypt.

### Participants and sampling

To be eligible for enrollment in the study, participants had to agree to participate in the study, aged 18 years old or older, on stable doses of medicine for at least 30 days from entering the study and diagnosed with IBS-D in accordance with Rome IV diagnostic criteria for IBS. The following cases were excluded from our study:

- Patients with severe lesions in major organs such as the heart, liver, and kidney, hematopoietic diseases, or tumors.
- Eating disorders.
- History of major abdominal surgery.
- History of neurological and mental illness.
- Usage of other treatments rather than medical treatment on a regular basis 2 weeks prior to randomization and throughout the study.
- Previous history of drug or alcohol abuse 6 months prior to randomization.
- Pregnant and lactating women.
- Patient reporting of adequate relief of their IBS symptoms the week preceding the randomization.
- The patient has too mild symptoms, obtaining less than 75 on the IBS-SSS.
- Currently participating in other clinical trials.
- Prior experience with acupressure or acupuncture therapy.

The sample size was estimated using Stata/SE14.2. Using IBS-SSS as a primary endpoint with a minimum clinical significance of 15% reduction in severity of symptoms [39] and based on the mean ± SD (273.94 ± 52.79) as reported in an earlier study [40]. It was calculated that 60 subjects (30 in each group) were needed to detect a difference between the two groups with a power of 90% at the 5% level of statistical significance. The sample size was increased to 70 to adjust for the potential dropout rate.

### Randomization and blinding

We employed simple randomization to equally assign the participants to either the AA group or SA group based on a randomization sequence that was generated using an online randomizer [41]. The randomization list was recorded and enclosed in a sequentially numbered, opaque envelope. Only the training researcher knew the group assignment and was blinded to the participants’ baseline and follow-up assessments throughout the study to avoid any selection bias. Conversely, participants and other relevant researchers (the outcome evaluator and data managers) and the statistician were blinded to the groups’ allocation.

### Study intervention and comparator

The participants in both groups received the same protocol of 120 min of training in two sessions by the training researcher, who is certified in acupressure from the faculty of physical education at Alexandria University, Egypt. The number of participants in each session ranged from two to seven based on their TCM diagnosis. The training was conducted on separate dates for both groups to prevent contamination. The first session was a discussion of the theoretical foundations of acupressure, which included an introduction to acupressure and the function(s) of each acupoint based on TCM philosophy. The second session was practical application, which included locating the selected acupoints and the details of the acupressure technique, followed by group practice. Each participant received a handout that was supplemented with graphics explaining how to self-locate and press the acupoints. The content of this handout was reviewed by five experts in medical-surgical nursing.

The intervention was performed by the participants at their homes. Participants were instructed to perform acupressure by themselves twice a day for 4 weeks, in a total of 56 sets. In addition, use the thumb or middle finger to self-press each acupoint using circular movements until they feel soreness, numbness, or heaviness, which are unique sensations interpreted as the flow of qi [42]. The force of pressing must be sufficiently strong but still within a comfortable range. A lubricant was used to decrease friction between the acupoints and the finger. Participants were verified for the correct technique, and any gaps identified were corrected.

Participants were instructed not to alter their lifestyle in any significant ways throughout the study (e.g., adopting a new diet or changing their exercise regimen). They could take the rescue medications as directed by their doctors if they have uncontrollable diarrhea or stomach discomfort. At the second training session, each participant received an additional structured diary to track their acupressure performance, frequency of stools, and requirement for any rescue medications.

Regarding the acupoint selection, all patients in the AA group were treated with basic acupoints. Moreover, according to their TCM diagnosis, additional acupoints were added (see Table 1). These points were selected after a review of the literature [22, 43, 44] and based on expert opinion. For the SA group, sham acupoints include sham Zhongwan (CV 12), sham Tianshu (ST 25), sham Sanyinjiao (SP 6), and sham Zusanli (ST 36). All the sham points are 2 cm outside and parallel to the actual points, which do not match any recognized acupoints and are thought to have no therapeutic effect.

To enhance the participants’ commitment, a weekly telephone call was done to remind participants in both groups to perform acupressure and to answer any questions. Follow-up evaluation was done by week 2 and week 4 by assessors blinded to participants’ group allocation by conducting an individualized face-to-face interview. The interview took about 10–15 min at each follow-up point.

**Table 1:** Active self-administered acupoints selection according to TCM diagnosis [22, 43, 44]

Syndrome	Acupoints
	Basic acupoints Zhongwan (CV 12), Tianshu (ST25) in alternation with Sanyinjiao (SP 6), Zusanli (ST 36)
**Spleen Qi Deficiency syndrome** − Loose stool − Loss of appetite − Fatigue − *Pale tongue with a thin white coat.* − *Weak pulse.*	+ Qihai (CV 6), Daheng (SP 15) in alternation with Yinlingquan (SP 9) Neiguan (PC 6)
**Spleen-yang deficiency syndrome** − Loose stool − Fatigue − Fear of cold in the abdomen − *Puffy pale tongue with slippery coating and slow fine pulse*	+Yinlingquan (SP 9), Daheng (SP 15)
**Yang Deficiency of the Spleen and Kidney syndrome** − Aversion to cold − Diarrhea in the morning − Fatigue − *Pale tongue*,* maybe swollen*,* with a thin white coat.* − *Pulse will be deep and slow*	+ Qihai (CV 6), Taixi (KI 3), Gongsun (SP 4)
**Large intestinal damp-heat syndrome** − Sticky and greasy stools, − Foul and smelly stool − Burning of the anus − *Red tongue with a yellow coat. The coat may also be greasy.* − *Wiry*,* rapid pulse*,* possibly slippery*	+Quchi (LI11), Neiting (ST44) in alternation with Shangqiu (SP5), Hegu (LI 4)
**Liver depression and spleen deficiency syndrome** − Diarrhea with abdominal pain − Fatigue − Occurring with or aggravating with emotional changes − *Thin tongue with white coat.* − *Wiry pulse.*	+ Taichong (LIV 3), Hegu (LI 4), in alternation with Yanglingquan (GB 34), Qimen (LIV 14)

CV = conception vessel; ST = stomach; SP = spleen; PC = pericardium; KI = kidney; LI = large intestine; LIV = liver; GB = gallbladder, *italic* represents disease signs (Tongue and Pulse)

### Ethical considerations

Before initiating the study, the Research Ethics Committee, Faculty of Nursing, Alexandria University, Egypt (IRB00013620, SN:2022-9-55) authorized the study protocol on September 13, 2022. It was registered on Clinicaltrials.gov under the registration number NCT05702255 on January 7, 2023. Following an explanation of the study’s purpose, each study participant signed informed consent. Participants’ data was kept confidential, and the study participants’ privacy was respected. To ensure confidentiality, all personal and medical information was stored securely in password-protected electronic files, while data collection sheets were kept in locked cabinets accessible only to the principal investigator. Additionally, participant identifiers were replaced with coded numbers to further protect their anonymity throughout the study process. Participants were informed that their participation was completely optional and that they might opt out of the study at any time without any harmful consequences. In the event of withdrawal, any data already collected from the participant would be excluded from the final analysis. This ensures that only data from participants who completed the study were analyzed.

### Measurements

A designed form was developed to collect participants’ characteristics, including gender, age, body mass index (BMI), patients’ physical activity status (classified as “physically inactive” if they did not exercise frequently or as “physically active” if they exercised at least once a week), following a specific diet, smoking status, IBS control medication, and their TCM diagnosis.

#### The Irritable Bowel Syndrome Symptom Severity Scale (IBS-SSS)

is as five-item measure of abdominal pain intensity and frequency, abdominal distension severity, the degree of dissatisfaction with bowel movements, and the interference of IBS with everyday life. On a 100-point visual analogue scale, subjects answer each question. There is a 500-point total score range. IBS can be classified as mild (75-<175), moderate (175–300), or severe (> 300) [45].

#### The IBS Adequate Relief Question (IBS-AR)

is a dichotomous single item that asks participants, “Over the past week, have you had adequate relief of your IBS symptoms?” [46]. It has been widely used to assess efficacy in IBS clinical trials [47, 48].

#### Stool frequency

was defined by the biweekly mean frequency of defecation. It was calculated from the patient’s self-report of the number of defecations per day.

#### The Bristol Feces Form Scale (BSFS)

the consistency of the stool was evaluated using the BSFS. the BSFS is an ordinal scale of stool form Type 1(hardest stool) to Type 7 (softest stool). Types 1 and 2 are abnormally hard stools form, Types 3, 4, and 5 are the most normal stool form, whereas Types 6 and 7 are regarded as excessively loose or liquid stool form. The BSFS has seven pictures that correspond to stool types to facilitate the recording of stool consistency [49].

#### The Hospital Anxiety and Depression Scale (HADS)

is a self-rating tool designed to evaluate psychological distress in non- psychiatric patients within a hospital medical outpatient clinic. It contains 14 items that contribute to two subscales: depression (HADS-D) and anxiety (HADS-A). Each statement was rated on a four-point rating scale (0 to 3); the total score for each subscale ranges from 0 to 21 [50]. There is no anxiety or depression if the score is ≤ 7, a score between 8 and 10 denotes mild anxiety or depression, between 11 and 15 suggests moderate anxiety or depression, and a score of 16 or more denotes severe anxiety or depression [51]. The Arabic version of HADS was used [52].

### Use of rescue medicine, compliance and adverse events

Participants were asked to report the use of rescue medicine, adherence to acupressure sessions, and any adverse events related to or not to acupressure during the study period. Adherence was calculated as a percentage of planned sessions, and a minimum of 80% was considered acceptable.

### Statistical analysis

To analyze the data, IBM SPSS version 25.0 was utilized. In descriptive statistics, percentages and frequencies were used to characterize qualitative variables, whereas means and standard deviations were used to characterize quantitative variables. Shapiro-Wilk and Kolmogorov-Smirnov tests were used to judge the normal distribution of numeric variables, and Levene’s test was used to evaluate the homogeneity of variance. For intergroup comparison, an independent sample t test was used for parametric data. Nonparametric tests (Chi-square test and Mann-Whitney) were used according to the categorical or continuous nature of the variables. A paired t test for parametric data and a paired Wilcoxon test for non-parametric data were used to analyze the intragroup comparison of the change from the base line to weeks 2 and 4. The McNemar chi-square test and the marginal homogeneity test, respectively, were used to determine the significance of changes in binary and multinomial variables. A *p*-value of less than 0.05 was set as the statistical significance threshold.

## Results

### Characteristics of the study participants

**Fig. 1:**
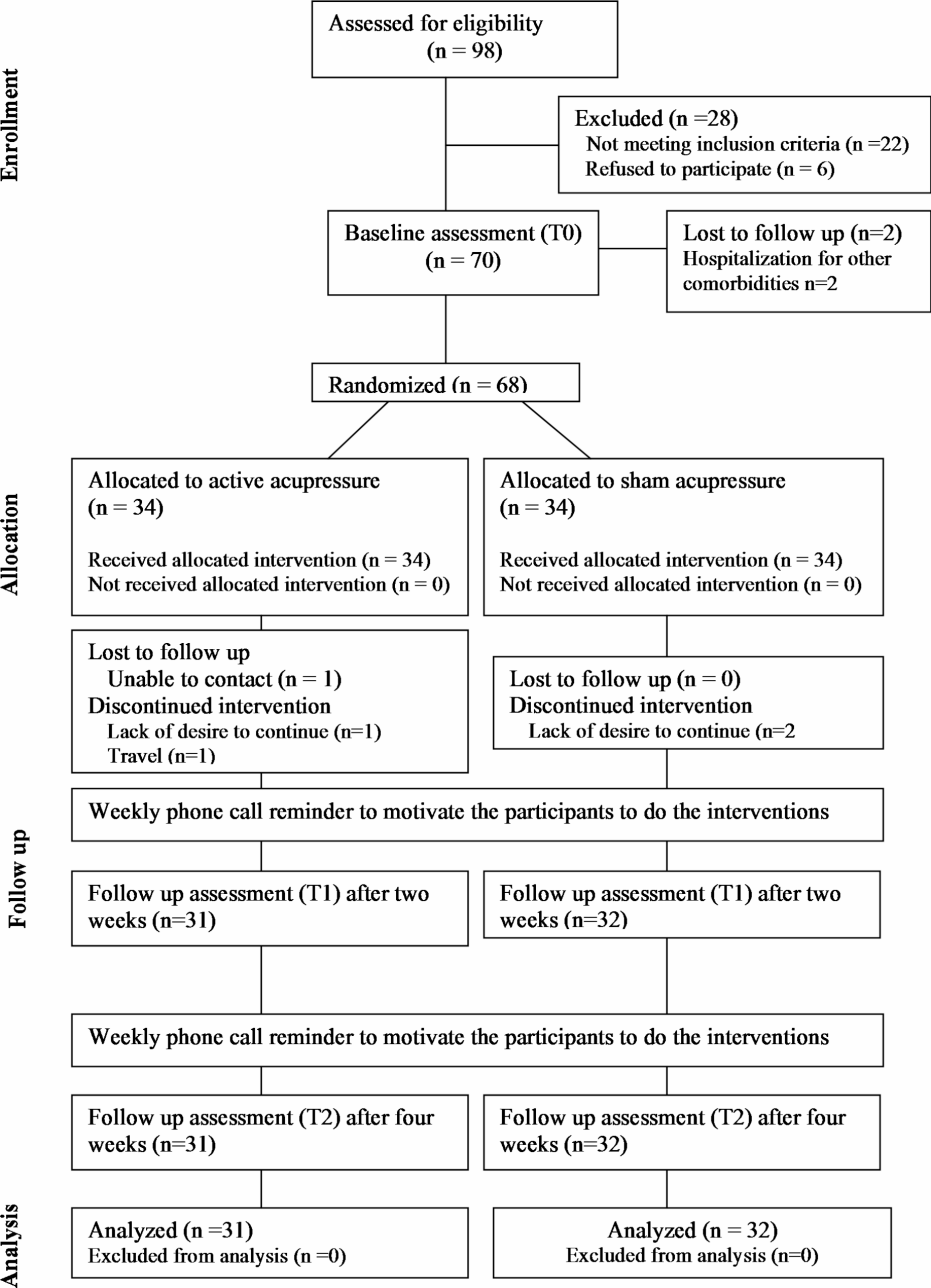
Flow diagram of the study

The study was conducted between February 5, 2023, and August 28, 2023, during which 98 participants were screened for eligibility. Of these, 22 participants were excluded for not meeting the inclusion criteria, and 6 declined to participate. After the baseline assessment, 2 participants were lost to follow-up, and the remaining 68 were randomized into two groups. In the end, 63 patients (AA group = 31, SA group = 32) completed the trial and were analyzed (see Fig. 1).

The reliability of the tools used in this study was evaluated to ensure consistency of the results. The IBS-SSS showed good reliability with a Cronbach’s alpha coefficient of 0.82. Similarly, the HADS-D and HADS-A demonstrated acceptable internal consistency with α = 0.77 and α = 0.85, respectively.

As shown in Table 2, the participants’ average age was 41.26 ± 7.37 in the AA group and 40.91 ± 9.1 in the SA group. Most participants in both groups were female, with 64.5% in the AA group and 68.8% in the SA group. The most common diagnoses in both groups were Spleen Qi Deficiency Syndrome and Spleen-Yang Deficiency Syndrome. In terms of smoking status, most participants were nonsmokers (67.7% in the AA group vs. 71.9% in the SA group). Most of the participants in the AA group (61.3%) reported no need for rescue drugs during the study period, compared to 40.6% in the SA group.

Regarding physical activity, 58.1% of the AA group and 43.75% of the SA group were inactive. Most patients in both groups had no other comorbid conditions (74.2% in the AA group vs. 65.6% in the SA group). The average BMI was 28.46 ± 2.44 in the AA group and 26.96 ± 3.17 in the SA group. Additionally, most participants were overweight (61.3% in the AA group vs. 56.25% in the SA group). The two groups were well-matched in terms of baseline demographic and clinical characteristics (*p* > 0.05 for all comparisons), except for BMI (*p* = 0.04).

**Table 2:** Socio-demographic and clinical characteristics among the study participants

Characteristics	Active acupressure group (*n* = 31)			Sham acupressure group (*n* = 32)		Test statistic (*p*)
	*n*	%		*N*	%	
**Age (X ±** ***SD*)**	**41.26 ± 7.37**			**40.91 ± 9.1**		t = 0.168(0.86)
< 40	16		51.6	17	53.1	
40-<50	10		32.3	9	28.1	
≥ 50	5		16.1	6	18.8	
**Gender**						
Male	11		35.5	10	31.2	X^2^ = 0.127(0.772)
Female	20		64.5	22	68.8	
**TCM diagnosis**						
Spleen Qi Deficiency syndrome	9		29.0	11	34.4	X^2^ = 2.13(0.757)
Spleen-yang deficiency syndrome	10		32.3	7	21.9	
Yang Deficiency of the Spleen and Kidney syndrome	4		12.9	4	12.5	
Large intestinal damp-heat syndrome	6		19.4	5	15.6	
Liver depression and spleen deficiency syndrome	2		6.5	5	15.6	
**Physical activity**						
Active	13		41.9	18	56.25	X^2^ = 1.29(0.25)
In-active	18		58.1	14	43.75	
**Follow therapeutic diet**						
Yes	19		61.3	22	68.8	X^2^ = 0.386(0.53)
No	12		38.7	10	31.2	
**Co-morbidity**						
Yes	8		25.8	11	34.4	X^2^ = 0.549(0.45)
No	23		74.2	21	65.6	
**Use of medicine**						
One	19		61.3	12	40.6	X^2^ = 2.75(0.25)
Two	7		22.6	12	37.5	
More than two	5		16.1	7	21.9	
**Smoking status**						
Smoker	10		32.3	9	28.1	X^2^ = 0.128(0.721)
Non-smoker	21		67.7	23	71.9	
**Body mass index (X ±** ***SD*)**	**28.46 ± 2.44**			**26.96 ± 3.17**		t = 2.09(0.04*)
Healthy weight	4		12.9	8	25	
Overweight	19		61.3	18	56.25	
Obese	8		|25.8	6	18.75	

X^2^: Chi Square test of independence, X^2FE^: Fissure exact test, **t**: independent sample t rest, * level of significance *p* ≤ 0.05

### Comparative effects of active and sham acupressure on study outcomes

Table 3 presents the comparisons of study outcomes between the groups at each time point, while Table 4 shows the outcomes within each group at T1 and T2 compared to T0.

### Primary outcomes of active acupressure compared to sham acupressure

At baseline and week 2, there were no statistically significant differences in IBS-symptom severity between the AA group (T0: 208.39 ± 39.01, T1: 205.32 ± 41.93) and the SA group (T0: 212.81 ± 39.57, T1: 210.31 ± 39.61), with most participants in both groups showing moderate symptom severity. However, by week 4, both groups experienced a significant reduction in symptom severity compared to baseline. Notably, the AA group had a significantly lower symptom severity score (181.12 ± 30.62) than the SA group (202.34 ± 47.68), with *p* = 0.04.

By week 2, both groups reported significant relief of IBS symptoms compared to baseline (AA group: 7 participants, 22.6%, *p* = 0.016; SA group: 5 participants, 15.6%, *p* = 0.063), though there were no significant differences between the groups at T1 (*p* = 0.494). By week 4, acupressure significantly contributed to adequate relief, with 21 participants (67.74%) in the AA group reporting relief, compared to 8 participants (25%) in the SA group, showing a statistically significant difference between the two groups (*p* = 0.032).

At baseline, there were no significant differences in stool frequency between the AA group (37.48 ± 5.04) and the SA group (38.06 ± 8.14) (*p* = 0.7). However, stool frequency significantly decreased in both groups throughout the intervention. By weeks 2 and 4, the AA group exhibited significantly lower stool frequency (T1: 31.68 ± 3.63; T2: 28.48 ± 2.44) compared to the SA group (T1: 34.28 ± 3.79; T2: 32.22 ± 2.88).

By week 2, 16.1% of participants in the AA group showed a significant shift toward normal stool form (*p* = 0.02), while the SA group did not show any significant change from baseline (*p* = 0.08). By week 4, both groups exhibited a significant shift toward normal stool form compared to baseline (AA group: 67.7%, *p* < 0.001; SA group: 21.9%, *p* = 0.013), with the difference between the two groups also being statistically significant (*p* < 0.001).

### Secondary outcomes of active acupressure compared to sham acupressure

At baseline, 29% of the AA group experienced mild anxiety (mean: 7.12 ± 1.23), compared to 21.9% experiencing mild anxiety and 6.2% experiencing moderate anxiety in the SA group (mean: 6.98 ± 1.55). Mild depression was present in 25.8% of the AA group (mean: 6.93 ± 1.18) compared to 21.9% in the SA group (mean: 6.96 ± 1.01). There were no significant differences between the groups at T0 and T1 for anxiety or depression. However, at T2, the AA group showed a significant decrease in anxiety (6.31 ± 1.43) and depression (6.2 ± 0.83) compared to the SA group (6.73 ± 1.98 and 6.83 ± 1.24, respectively).

By week 2, 15 participants (48.4%) in the AA group reported need for rescue drugs, compared to 13 participants (40.6%) in the SA group. By week 4, the AA group showed a significantly lower need for rescue medication (27 participants, 77.4%) compared to the SA group (10 participants, 31.3%). Over time, the AA group exhibited a statistically significant reduction in the need for rescue medication (*p* = 0.021), whereas the SA group did not (*p* = 0.508).

Additionally, the compliance rate for all study participants was over 80%. Regarding adverse events, two participants (one from the AA group and one from the SA group) reported redness, while one participant in the AA group experienced a mild headache. These cases were reported to the treating physician and were successfully managed, with symptoms resolving.

**Table 3:** Intergroups comparisons of the study outcomes across different time points

Outcomes	Measurement time	Active acupressure group (*n* = 31)	Sham acupressure group (*n* = 32)	Test statistics(*p*)
		No. (%)	No. (%)	
**IBS-SSS**	**T0 (X ±** ***SD)***	**208.39 ± 39.01**	**212.81 ± 39.57**	t=-0.447(0.657)
	Mild	6(19.4)	6(18.8)	
	Moderate	25(80.6)	26(81.3)	
	**T1(X ± SD)**	**205.32 ± 41.93**	**210.31 ± 39.61**	t=-0.486(0.629)
	Mild	8(25.8)	7(21.9)	
	Moderate	23(74.2)	25(78.1)	
	**T2(X ± SD)**	**181.12 ± 30.62**	**202.34 ± 47.68**	t=-2.09(0.04*)
	Mild	14(45.2)	12(37.5)	
	Moderate	17(54.8)	20(62.5)	
**IBS-AR**	**T1**			
	Yes	7(22.6)	5(15.6)	X^2^ = 0.494 (0.53)
	No	24(77.4)	27(84.4)	
	**T2**			
	Yes	21(67.74)	8(25)	X^2^ = 4.72 (0.032*)
	No	10(32.26)	24(75)	
**Stool frequency**	**T0(X ± SD)**	**37.48 ± 5.04**	**38.06 ± 8.14**	U = 468.5(0.7)
	**T1(X ± SD)**	**31.68 ± 3.63**	**34.28 ± 3.79**	t= -2.77(0.007*)
	**T2(X ± SD)**	**28.48 ± 2.44**	**32.22 ± 2.88**	t=-0.53(< 0.001*)
**BSFS**	**T0**			
	Loose (6–7)	31(100%)	32(100%)	U = 468.5(0.61)
	**T1**			
	Normal (4–5)	5(16.1)	2(6.3)	U = 432(0.29)
	Loose (6–7)	26(83.9)	30(93.8)	
	**T2**			U = 261(< 0.001*)
	Normal (4–5)	21(67.7)	7(21.9)	
	Loose (6–7)	10932.3)	25(78.1)	
**HADS-A**	**T0(X ± SD**)	**7.12 ± 1.23**	**6.98 ± 1.55**	U = 415.5(0.24)
	No	22(71)	23(71.9)	
	Mild	9(29)	7(21.9)	
	Moderate		2(6.2)	
	**T1(X ± SD**)	**6.9 ± 1.3**	**6.96 ± 1.55**	U = 402.5(0.16)
	No	23(74.2)	25(78.125)	
	Mild	8(25.8)	6(18.75)	
	Moderate		1(3.125)	
	**T2(X ± SD)**	**6.31 ± 1.43**	**6.73 ± 1.98**	U = 324(0.015*)
	No	27(87.1)	26(81.25)	
	Mild	4(12.9)	6(18.75)	
**HADS-D**	**T0(X ± SD**)	**6.93 ± 1.18**	**6.96 ± 1.01**	U = 493.5(0.97)
	No	23(74.2)	25(78.1)	
	Mild	8(25.8)	7(21.9)	
	**T1(X ± SD**)	**6.87 ± 1.15**	**6.88 ± 0.98**	U = 452 (0.72)
	No	25(80.65)	25(78.1)	
	Mild	6(19.35)	7(21.9)	
	**T2(X ± SD)**	**6.2 ± 0.83**	**6.83 ± 1.24**	U = 254.5(0.02*)
	No	29(93.54)	24(75)	
	Mild	2(6.45)	8(25)	
**Use of rescue medicine**	**T1**			
	Yes	15(48.4)	13(40.6)	X^2^ = 0.34(0.53)
	No	16(51.6)	19(59.4)	
	**T2**			
	Yes	7(22.6)	22(68.8)	X^2^ = 0.601(0.043)
	No	27(77.4)	10(31.3)	

IBS-SSS: Irritable Bowel Syndrome Symptom Severity Score, BSFS: Bristol Stool Form Scale, HADS-A: Hospital Anxiety and Depression Scale Anxiety, HADS-D: Hospital Anxiety and Depression Scale Depression, T0: Baseline, T1: Week 2, T2: Week 4, t: independent sample t test, U = Mann Whitney U test, X2: Chi square test, *: significance *p* ≤ 0.05

**Table 4:** Intragroup comparisons of the study outcomes between different time points

Outcomes	Active acupressure group (*n* = 31)	Sham acupressure group (*n* = 32)
	Test statistics (*p*)	Test statistics (*p*)
**IBS-SSS**		
T1-T0	t=-1.49(0.14)	t=-1.5(0.143)
T2-T0	t = 2.-6(0.014*)	t=-2.27(0.03*)
**IBS-AR**		
T1-T0	X^2MC^ = 5.14(0.016*)	X^2MC^ = 3.2(0.063)
T2-T0	X^2MC^ = 17.05(< 0.001*)	X^2MC^ = 6.12(0.008*)
**Stool frequency**		
T1-T0	Z=-4.313(< 0.001*)	Z=-2.47 (0.013*)
T2-T0	Z=-4.86(< 0.001*)	Z =-3.96(< 0.001*)
**BSFS**		
T1-T0	MH = 30(0.02*)	MH = 16(0.08)
T2-T0	MH = 119(< 0.001*)	MH = 64(0.013*)
**HADS-A**		
T1-T0	Z=-1.35(0.16)	Z=-1.29(0.12)
T2-T0	Z=-3.6(< 0.001*)	Z=-1.36(0.08)
**HADS-D**		
T1-T0	Z=-1.55 < 0.45)	Z=-1.2 (0.87)
T2-T0	Z=-4.13(< 0.001*)	Z=-0.89(0.24)
**Use of rescue medicine**		
T2-T1	X^2MC^ = 4.9(0.021*)	X^2MC^ = 0.44(0.50)

IBS-SSS: Irritable Bowel Syndrome Symptom Severity Score, BSFS: Bristol Stool Form Scale, HADS-A: Hospital Anxiety and Depression Scale Anxiety, HADS-D: Hospital Anxiety and Depression Scale Depression, T0: Baseline, T1: Week 2, T2: Week 4, t: paired sample t test, Z: Wilcoxon Signed Rank Test, X^2MC^: chi square for MCNemar’s test, MH: Marginal homogeneity test, *: significance *p* ≤ 0.05

## Discussion

We conducted an RCT that investigated the effects of acupressure on IBS-D patients, dividing participants into two groups—one receiving AA and the other receiving SA. Then, we measured the effect of the interventions on primary and secondary outcomes among patients. The primary outcomes investigated include symptom severity, adequate relief, stool frequency, and consistency. Secondary outcomes like psychological wellbeing, use of rescue medications, and adverse events were also examined. We measured the effect of active versus sham acupressure on these outcomes at three time points: baseline (T_0_), week 2 (T1), and week 4 (T2).

### The role of acupressure in symptoms severity and adequate relief report among IBS-D patients

Our RCT revealed a gradual and significant decrease in the severity of symptoms among patients who received AA compared to those who received SA. Notably, both groups experienced some symptom relief over time, but the AA group demonstrated significantly better outcomes in both symptom severity and adequate relief. These findings highlight the promising potential of acupressure as part of the treatment plan for IBS-D patients.

Acupressure’s therapeutic effects might be attributed to the fact that acupressure may modulate the autonomic nervous system, reducing stress and enhancing gastrointestinal functions. The greater improvement observed at the four-week assessment suggests that the therapeutic effects of acupressure might require consistent application over time to achieve optimal benefits. This gradual improvement aligns with the holistic and cumulative nature of acupressure’s therapeutic mechanisms.

According to Zhang et al. (2022), acupressure may alleviate symptoms of IBS by regulating brain-gut peptides, modifying cerebral connectivity and activity, enhancing neuroendocrine functions and mental state, and reducing inflammation and bowel hypersensitivity [16].

According to the central nervous system’s visceral hyperalgesia theory, Wal et al. (2022) demonstrated how acupressure may affect the visceral system by triggering the somatic system. Applying pressure to this area may help reduce the symptoms of IBS, including bloating, constipation, diarrhea, bloody stools, stomach pain, and lack of appetite [53]. Moreover, Xing et al. (2013) found that yin and yang in the spine may be balanced by acupressure at Jiaji points, which would promptly alleviate IBS symptoms [54].

Several studies support the efficacy of the acupoints’ therapy in managing IBS and other gastrointestinal disorders. For instance, a study by Huang et al. (2021) demonstrated significant improvements in IBS symptoms and QOL with regular acupuncture sessions [55]. Yang et al. (2023) revealed in their study that acupuncture significantly reduces IBS symptoms severity and improves patient QOL without any adverse effects [56]. Also, the studies of Guo et al. (2020) and Pei et al. (2020) revealed that acupuncture improves patients’ overall symptoms, decreases the recurrence rate, improves the total effective rate, and reduces the pain level of patients with IBS [57, 58].

On the other hand, Yang et al. (2022) found no significant difference in anxiety between acupuncture and sham acupuncture (SA) among IBS-D patients [59]. This suggests that the psychological benefits observed in the current study might be influenced by patients’ expectations and beliefs about the efficacy of acupressure, which could be significantly shaped by cultural factors. In societies where Chinese medicine is widely accepted, patients may have higher expectations regarding the effectiveness of such treatments.

Interestingly, our study also revealed notable decrease in symptom severity in the SA group, though these were less pronounced than in the AA group. At week 2, the SA group demonstrated a trend toward symptom relief (15.6%, *p* = 0.063), which became more apparent by week 4, with 25% of participants reporting adequate relief. While statistically less significant, these results suggest the influence of non-specific effects, such as the placebo effect.

The tactile stimulation and structured therapeutic interaction in the SA protocol likely contributed to this improvement, creating a sense of care and reducing stress. This aligns with the theory that even general tactile stimulation can partially activate somatic-visceral pathways, as suggested by Wal et al. (2022) [53]. While the effects of SA do not fully mimic those of active acupressure, they may still induce mild autonomic or neuroendocrine responses, leading to transient relief. These findings emphasize the need for further research to disentangle the mechanisms of acupoint-specific effects from general tactile stimulation and placebo responses.

These findings of this study contradict those of Forbes et al. (2005) and Lowe et al. (2017), who found that, despite improvements in both the active and sham acupuncture groups, there was no statistically significant difference between the two groups at the three-month follow-up [60, 61]. This could be explained by sham needling having significant physiological effects, such as enhanced endorphin activation.

### The role of acupressure in regulating stool consistency and frequency among IBS-D patients

While our results indicate that AA provided superior outcomes in stool frequency and stool form, the moderate improvements observed in the sham SA group warrant further exploration of non-specific therapeutic mechanisms. Both groups experienced significant reductions in stool frequency and shifts toward normal stool form by week 4, but the AA group demonstrated a distinct advantage. These findings suggest that precise acupoint stimulation enhances therapeutic outcomes, potentially by leveraging specific neuroendocrine and autonomic pathways more effectively than general tactile interaction, thus regulating gut motility and reducing gastrointestinal symptoms [62]. The study indicates that acupressure therapy, when used regularly, can gradually improve bowel function by enhancing neural pathways and endocrine responses, aligning with TCM principles.

In the SA group, the improvements in stool frequency and form may be attributed to non-specific effects, such as tactile stimulation and patient-provider interaction. The act of applying pressure, even without targeting specific acupoints, might activate somatosensory pathways that contribute to symptom relief. This aligns with previous evidence suggesting that therapeutic touch and the placebo effect can positively influence gastrointestinal function. Furthermore, the psychological benefits of structured care, including reduced stress and heightened perceptions of support, could have played a role in the SA group’s outcomes.

Our findings are supported by the recent work of Zhao et al. (2024) who investigated the effect of true acupuncture versus sham acupuncture on refractory IBS with 94% of their study sample was IBS-D, their study reveals that individuals who had true acupuncture experienced a greater improvement in regular stools than those who got sham acupuncture. Additionally, the QOL and anxiety symptoms of the individuals in the acupuncture group improved [63]. Zhu et al. (2018) highlighted that IBS-D reported decreased passage of the flatus and bloating after acupuncture [64], which supports our findings. In addition, Khan-Mohammadi et al. (2023) showed in their study that patients undergoing coronary artery bypass surgery had a significant improvement bowel movements and stool after applying acupressure indicating that acupressure can positively affect intestinal function [65]. Additionally, Go & Park (2020) found that auricular acupressure helped IBS patients with abdominal pain, discomfort, loose stools, and stress [66]. A study by Kong et al. (2018) revealed that auricular acupressure significantly reduces the incidence rates of nausea, vomiting, and diarrhea among gastric cancer patients [67]. These studies reinforce the notion that acupressure can modulate gastrointestinal function, providing a plausible explanation for the improvements seen in IBS-D patients in the current trial.

### The role of acupressure in maintaining psychological well-being among IBS-D patients

Our RCT revealed a significant prevalence of anxiety and depression among the AA group. The results reveal that psychological wellbeing among the AA group was significantly maintained compared to the SA group, as there was a significant decrease in anxiety levels and depressive symptoms among the AA group. Our analysis also revealed that improvements were more pronounced in the fourth week compared to the second week, suggesting a cumulative effect of acupressure over time. This time-dependent improvement underscores the importance of long-term adherence to the therapy to achieve optimal benefits.

These results are supported by the earlier results of Kabra & Nadkarni (2013) who reported a high prevalence of depression (37.1%) and anxiety (31.4%) in IBS patients [68]. Likewise, a prior comprehensive review found that IBS patients had considerably greater levels of depression and anxiety, which could worsen their symptoms [69].

According to Lee & Frazier, (2011) acupressure may induce such improvement by regulating the yin-yang balance of energy and blood flow and promoting body fluid circulation and physiological balance [70]. Furthermore, acupoint stimulation increases serotonin and endorphin synthesis, which reduces anxiety which are known to enhance mood and reduce pain perception [63].

This result gives insight that acupressure provides a holistic approach to the treatment of IBS-D patients due to its impact on both physical and psychological aspects of health. In the context of IBS-D, where psychological factors play a significant role in symptom exacerbation, addressing both the mind and body through interventions like acupressure could lead to more comprehensive and effective management strategies. This approach aligns with the bio-psychosocial model of health, which emphasizes how social, psychological, and biological factors interact to treat illness.

The beneficial effects of acupressure on psychological wellbeing and anxiety have been supported by other studies as well. For instance, a meta-analysis by Wang et al. (2022) also confirmed the positive effects of acupuncture on managing anxiety and depression of patients with IBS-D [71]. Rani et al. (2020) found that acupressure significantly reduced anxiety and depression in patients with chronic pain conditions [72]. Similarly, Xie et al. (2024) reported that acupressure effectively alleviated symptoms of depression and anxiety in cancer patients [73]. A meta-analysis by Au et al. (2015) also demonstrated a positive effect of acupressure on relieving anxiety across 27 studies [74]. While these findings support the idea that acupressure can positively influence psychological health, it is important to consider other contributing factors. For instance, the holistic nature of the treatment and the therapeutic interaction between the practitioner and the patient might reduce anxiety and enhance feelings of wellbeing, independent of the specific effects of acupressure itself.

### The role of acupressure in reducing the use of rescue medicine among IBS-D patients

Our RCT found a significant reduction in the use of rescue medications among the AA group compared to the SA group. This finding is expected since acupressure stimulates specific points that help regulate gastrointestinal motility and reduce visceral hypersensitivity, which are common issues in IBS-D patients [75]. Additionally, therapeutic touch and focused attention during AA, or even SA sessions, acupressure sessions might enhance the placebo effect, leading to greater symptom relief and a reduced need for rescue medications [71]. This finding is particularly important for patients who experience side effects from conventional medications or prefer natural treatment methods. Healthcare providers could consider integrating acupressure into standard IBS-D management protocols, potentially improving patient outcomes and satisfaction.

This finding is the case in the study by Pei et al. (2020), which found that patients who underwent AA exhibited minimal reliance on rescue medications [57]. Furthermore, Mróz et al. (2022) found that acupressure-induced improvements in physiological balance, coordination, and bodily fluid circulation were linked to a reduction in the use of rescue and over-the-counter medications in patients with gastrointestinal disorders [76], which supports our finding. Overall, our RCT suggests that acupressure is a viable treatment option that produces improvement in the physio-psychological symptoms of IBS-D in a cost effective and friendly manner.

### Implications for clinical nursing practice

These findings have important implications for clinical nursing practice. Given the chronic and often refractory nature of IBS-D, implementing acupressure as a nursing intervention could provide a non-pharmacological option to manage the physical and psychological symptoms associated with the condition. The nursing care plan for IBS-D should be tailored to include AA as a pivotal intervention. Nurses can be trained to administer or teach patients AA techniques, providing an additional tool to manage symptoms and improve patient outcomes. This approach not only addresses symptom management but also aligns with holistic nursing principles, promoting patient autonomy and potentially enhancing the overall QOL for individuals with IBS-D.

For clinicians, our RCT emphasized the importance of exploring complementary therapies in the management of IBS-D. The marked reduction in rescue medication usage indicates that AA could serve as an effective adjunctive treatment. Clinicians should consider integrating acupressure into their therapeutic arsenal, especially for patients who may be seeking alternatives to traditional pharmacological treatments due to side effects or insufficient symptom control. This integration can lead to a more comprehensive and individualized approach to care, addressing both the physiological and psychological aspects of IBS-D.

The study’s findings also encourage a broader reevaluation of treatment protocols for IBS-D within clinical settings. Health care providers might consider routine assessments of acupressure efficacy as part of follow-up appointments, adjusting treatment plans based on patient responses. Additionally, the incorporation of such non-invasive and cost-effective therapies could lead to reduced healthcare costs and resource utilization, presenting a compelling case for wider adoption and integration into standard clinical practice.

### Strengths and limitations

This study has several strengths. First, the randomized controlled trial (RCT) design enhances the reliability of the findings by comparing the active acupressure (AA) group with a sham acupressure (SA) group, ensuring that the results are attributable to the intervention. Second, the use of both primary and secondary outcomes—including symptom severity, stool consistency, and psychological well-being—provides a comprehensive assessment of the intervention’s effects. Third, the integration of a patient-friendly, non-pharmacological treatment option aligns with holistic nursing care, empowering patients to manage their symptoms independently, which is a cost-effective and accessible approach.

Despite its strengths, the study has some limitations. The sample size of 63 participants, though adequate for detecting significant differences, may limit the generalizability of the findings to broader populations. Additionally, the study’s short duration (four weeks) raises questions about the long-term effectiveness of the intervention, which future research should address. The study also relied on patient-reported outcomes, which could introduce subjective biases. Although the sham control group helps mitigate the placebo effect, blinding may not have been entirely effective, as some participants might have discerned the difference between active and sham acupressure.

The study’s reliance on a single healthcare setting also limits the generalizability of its findings. Future research should explore the intervention’s applicability across diverse healthcare environments and patient populations. Another limitation is the potential variability in participants’ adherence to the acupressure protocol, which could affect the consistency of the results. Despite efforts to monitor adherence, participants’ self-reported compliance may have introduced inaccuracies.

## Conclusion

This study suggests that self-administered active acupressure is a viable and beneficial non-pharmacological intervention for managing diarrhea-predominant irritable bowel syndrome (IBS-D). It significantly reduces symptom severity, improves stool consistency and frequency, and enhances psychological well-being, offering a holistic approach to symptom management. The findings highlight the potential of incorporating acupressure into standard care protocols for IBS-D, particularly as a cost-effective, patient-friendly option that reduces the reliance on medications. Future research should explore the long-term effects and scalability of this intervention across diverse patient populations.

## Data Availability

The datasets and materials of the current study are available from the corresponding author on reasonable request.

## Abbreviations

IBS: Irritable Bowel Syndrome
IBS-D: Diarrhea-Predominant Irritable Bowel Syndrome OR IBS with Diarrhea
FGID: Functional Gastrointestinal Disorder
QOL: Quality Of Life
AA: Active Acupressure
SA: Sham Acupressure
RCTs: Randomized Controlled Trials
T0: Baseline
T1: Week 2
T2: Week 4
IBS-SSS: The Irritable Bowel Syndrome Symptom Severity Scale
IBS-AR: The IBS Adequate Relief Question
BSFS: The Bristol Stool Form Scale
HADS: The Hospital Anxiety and Depression Scale
HADS-A: The Hospital Anxiety and Depression Scale- anxiety
HADS-D: The Hospital Anxiety and Depression Scale -Depression
